# Editorial: Approaching human intelligence through chemical systems: development of unconventional chemical artificial intelligence

**DOI:** 10.3389/fchem.2023.1332647

**Published:** 2023-11-09

**Authors:** Pier Luigi Gentili, Konrad Szaciłowski, Andrew Adamatzky

**Affiliations:** ^1^ Department of Chemistry, Biology, and Biotechnology, Università degli Studi di Perugia, Perugia, Italy; ^2^ Academic Centre for Materials and Nanotechnology, AGH University of Kraków, Kraków, Poland; ^3^ Unconventional Computing Laboratory, University of the West of England, Bristol, United Kingdom

**Keywords:** neuromorphic engineering, synthetic cells, molecular networks, chemical Robots, molecular cybernetics, molecular computing, supramolecular chemistry, systems chemistry

Artificial intelligence (AI) and robotics are having a soaring impact on our societies. They pervade our lives (Russell and Norvig, 2010; Kurzweil, 2014; Mitchell, 2019). There are two principal strategies for developing AI: one relies on current electronic computers or special-purpose hardware and consists of writing human-like intelligent software; the other is neuromorphic engineering in hardware. This Research Topic presents a brand-new strategy that was proposed for the first time about 10 years ago, in the early 2010s (Stojanovic, 2011; Gentili, 2013; Hagiya et al., 2014): it consists of using Molecular, Supramolecular, and Systems Chemistry in wetware (i.e., in fluid solution) to mimic some performances of human intelligence. This brand-new strategy has been called Chemical Artificial Intelligence (CAI) (Gentili, 2013) or Molecular Cybernetics (MC) (Murata et al., 2022). The final and ambitious purpose of CAI and MC is the implementation of Chemical Robots. Chemical Robots are intended to be autonomous cell-like structures with micrometric dimensions or so, which can reproduce some intelligent human behavior. Chemical Robots will allow humans to “colonize” the molecular world. Its colonization will provide tools to effectively combat all those pernicious macroscopic phenomena that emerge from the microscopic world, such as diseases, aging, and environmental pollution (Gentili P. L., 2021). The success of this alluring research line will require a productive interdisciplinary collaboration among chemists, biotechnologists, biologists, neuroscientists, cognitive scientists, computer scientists, physicists, engineers, and philosophers. This is the reason why this Research Topic has involved four distinct journals: Frontiers in Chemistry, Frontiers in Bioengineering and Biotechnology, Frontiers in Physics, and Frontiers in Robotics and AI.

Two contributions of this Research Topic outline the perspectives of CAI. They propose the first knowledge map for CAI, reporting its paradigms, which are the elements of the human nervous system; its domains, which are molecular, supramolecular, and systems chemistry; and finally, the problems CAI can face. In their first perspective, Gentili and Stano show that molecular and supramolecular chemistry can mimic some primary sensory, computing, communicating, and working actions of the human nervous system. In their second perspective, Gentili and Stano demonstrate that systems chemistry allows the design of more sophisticated modules to develop Chemical Robots. Chemical Robots should become capable of making rational decisions in environments dominated by uncertainty, partiality, and relativity of truth. These capabilities require the implementation of Fuzzy logic and Bayesian inference through molecules and their chemical reactions.

A promising strategy to encode Fuzzy sets and process Fuzzy logic is proposed by Gentili and Perez-Mercader. It is based on chemical micro-heterogeneity. Microheterogeneity refers to systems that are heterogeneous at the microscopic level. The heterogeneity can be at the level of single molecules (i.e., intra-entities) and/or inter-entities. It is intra-entities when it involves distinct conformers of a compound embedded in a homogeneous micro-environment. It is inter-entities when it is due to a complex mixture of different chemical compounds. Any micro-heterogeneous system can be exploited to encode a context-dependent Fuzzy set. This is the preliminary step for implementing Bayesian inference (Gentili P. L., 2021).

Networks constructed through nodes that are molecular Fuzzy sets allow to process Fuzzy logic. Gentili and Stano propose an avenue that is based on Synthetic Biology. The cross-talk between a couple of two-component signalling systems (each constituted by a sensor, a response regulator, and a gene) engrafted into Synthetic Cells originates a minimal network to process Fuzzy logic. The fundamental ingredients are the Fuzzy proteins, i.e., proteins that exist as collections of conformers, capable of showing multi-responsive and context-dependent behavior.

Sahoo and Baitalik present an alternative strategy for processing Fuzzy logic through molecules, which is hybrid because it is based on wetware and software. This hybrid strategy requires smooth analog input-output relationships of macroscopic variables, which can be modelled by building Fuzzy Logic Systems (Gentili, 2018). They apply this strategy to the anion and cation sensing power of a terpyridyl-imidazole-based receptor, whose responses also allow the implementation of multiple-configurable Boolean logic functions.

Bose et al. demonstrate that networks of interacting chemical oscillators are promising candidates for implementing the Chemical Robots’ brains. Such networks are valuable for recognising variable patterns, such as the patterns and symptoms in medical diagnosis. The authors propose their use for predicting the response of patients affected by myeloma to treatment with specific drugs based on their genetic profiles.

Spukti and Schnauss highlight the role that the protein actin can play in CAI. Actin, being part of the cytoskeleton, is capable of polymerizing, self-assembling, self-healing, and forming various bundle structures. Actin filaments are conductive to ionic currents, and mechanical and voltage solitons. The authors propose a procedure to prepare stable methyl cellulose-based actin aster networks, which will be relevant for computing purposes.

Vallverdú et al. present hormonal computing as an emerging field that explores incorporating hormone-based mechanisms into AI, neuromorphic engineering, and other computational systems. The endocrine system secretes hormones (i.e., chemical signals) that coordinate responses to stimuli in a specific manner. Hormones can communicate with certain body parts equipped with the necessary receptors to interpret and react to their signals. The dynamic, decentralized, and multifunctional properties of the hormonal system allow the design of innovative computing systems.

Finally, the last two contributions of this Research Topic face epistemological issues related to the development of CAI. Stano and Damiano advocate that trying to obtain intelligent chemical systems from scratch and in wetware can help understand intelligence and life. Life, conceived as a process, is a process of cognition. Therefore, a significant contribution to CAI will come from Synthetic Biology in general, and more specifically, from Synthetic Cells. Hence, Gentili and Stano propose how to quantitatively monitor the advancements in the technology of Synthetic Cells by determining their complexity degree through three approaches: 1) the reductionist, 2) the mesoscopic, and 3) the systemic one. A deeper understanding of intelligence and life will contribute to formulating more realistic models of Complex Systems and help humanity face the global challenges of this century (Gentili P. L., 2021).
